# Recent applications of the divinylcyclopropane–cycloheptadiene rearrangement in organic synthesis

**DOI:** 10.3762/bjoc.10.14

**Published:** 2014-01-16

**Authors:** Sebastian Krüger, Tanja Gaich

**Affiliations:** 1 Institut für Organische Chemie, Leibniz Universität Hannover, Schneiderberg 1B, 30167 Hannover, Germany

**Keywords:** cycloheptadiene, divinylcyclopropane rearrangement, domino reactions, natural products, total synthesis, vinylcyclopropane–carbaldehyde rearrangement

## Abstract

This review summarizes the application of the divinylcyclopropane–cycloheptadiene rearrangement in synthetic organic chemistry. A brief overview of the new mechanistic insights concerning the title reaction is provided as well as a condensed account on the biological relevance of the topic. Heteroatom variants of this rearrangement are covered briefly.

## Introduction

**Scheme 1:**
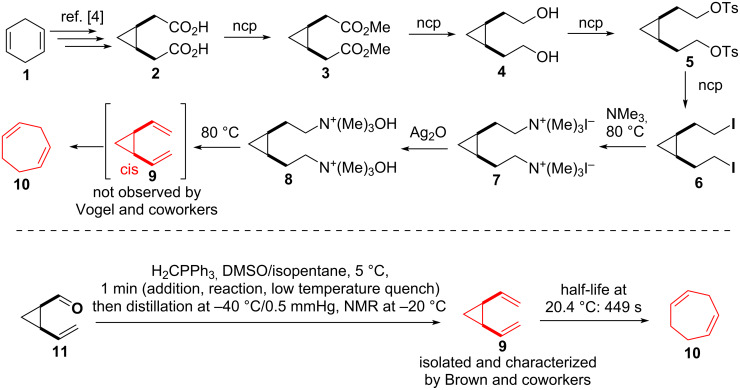
Vogel’s first approach towards the divinylcyclopropane rearrangement [4] and characterization of *cis*-divinylcyclopropane by Brown.

The first documented divinylcyclopropane–cycloheptadiene rearrangement dates back to 1960 occuring during studies of Vogel and coworkers [1–2] on the thermal rearrangement of small carbocycles. Although the desired *cis*-divinylcyclopropane (**9**) (see Scheme 1) could not be isolated using the depicted synthetic route (see **1** to **10)**, as **9** readily rearranged under the final Hofmann elimination conditions, the resulting cycloheptadiene **10** was described as well as the rearrangement of *trans*-divinylcyclopropane taking place at 200 °C. The elusive *cis*-divinylcyclopropane (**9**) was characterized ten years later by Brown and coworkers [3] using a low temperature and very short-timed Wittig reaction between *cis*-vinylcarbaldehyde **11** and methylenetriphenylphoshorane.

This review summarizes the recent synthetic applications of the divinylcyclopropane–cycloheptadiene rearrangement starting in 1991. Earlier classic syntheses of sesquiterpenes are described to underline the synthetic power in the total synthesis of sesquiterpenoid natural products. The title reaction has been subject to previous reviews [5–6], partial overlap with the content of this review to other reviews concerning different topics exist [7–10]. The divinylcyclopropane–cycloheptadiene rearrangement will be abbreviated as DVCPR in the following. The divinylcycloproane moiety and the resulting cycloheptadiene will be highlighted in red throughout. The Buchner ring expansion [11–12] as a special case of the DVCPR is not part of this review. The related vinylcylopropane–cyclopentene rearrangement has been reviewed elsewhere [13–14].

## Review

### Mechanistic considerations

**Scheme 2:**
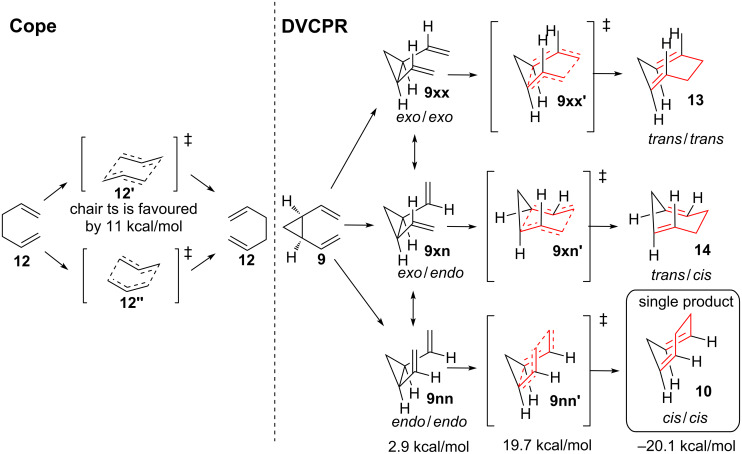
Transition states for the Cope rearrangement and the related DVCPR. Ts = transition state.

**Transition state**. Although the *cis*-divinylcyclopropane rearrangement is in fact a tethered version of the Cope rearrangement, it has to be noted that the preference of transition states (chair/boat) is opposite. Whereas the Cope rearrangement of hexa-1,5-diene **12** usually proceeds through chair-like transition state **12'** (see Scheme 2) and not through the energetically disfavoured boat-transition state **12''** [15], the DVCPR only proceeds via boat-like transition state **9nn'** where both vinyl-moieties are in the *endo*-orientation reagarding the cyclopropane [16]. The other transition states (**9xx'**/**9xn'**) would result in a cycloheptadiene with at least one *E*-configured double bond (**13**/**14**) after a hypothetical DVCPR, which can be regarded as inaccessible. Calculations revealed that the preferred orientation for *cis*-divinylcyclopropane is *exo*/*exo ***9xx**. The first rotation of a vinyl-moiety into the *endo*-orientation requires 0.8 kcal/mol giving **9xn**, the *endo*-orientation of both vinyl-moities requires 2.9 kcal/mol represented as **9nn** [17–19]. The necessary energy for the only possible transition state **9nn'** results in 19.7 kcal/mol, which is in good agreement with the experimental values of von Doering [20–21]. The rearranged cycloheptadiene **10** is favoured by −20.1 kcal/mol compared to the corresponding *cis*-divinylcyclopropane (**9**). Calculations have been carried out for *cis*-divinylheterocyclopropanes including nitrogen, oxygen, sulfur and phosphorus substitution [22], as well as *cis*-1,2-cyclobutanes [19,23]. Earlier calculations have been carried out for mono-heteroatom substitution in the vinyl moiety [24–25].

Transition state **9nn'** concludes that only *cis*-divinylcyclopropane undergoes the desired rearrangement, whereas *trans*-divinylcyclopropane should not react.

**Scheme 3:**
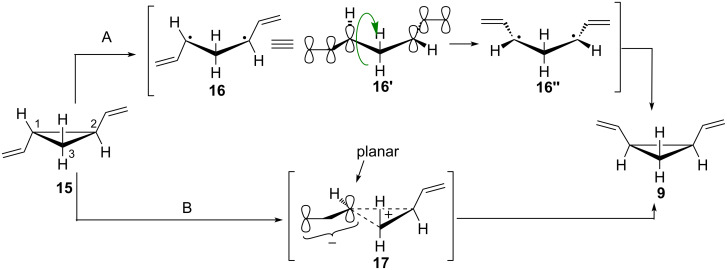
Two possible mechanisms of *trans*-*cis* isomerizations of divinylcyclopropanes.

***Trans-cis *isomerization.** Nevertheless*, trans*-divinylcyclopropane **15** (see Scheme 3) can be used in the DVCPR, as it undergoes isomerization to the desired *cis*-isomer **9** at elevated temperature (≈200 °C [1–2], lowered for more conjugated systems). The isomerization pathways have been suggested to proceed either via the formation of intermediate diradical-species (pathway A, Scheme 3) [16,20,26–27] or through one-center epimerization (pathway B) [28–29]. Following pathway A, the C1–C2 bond of **15** is cleaved homolytically to give diradical **16.** The two radicals are stabilized as allylic radicals (depicted as **16'**), rotation around the C1–C3 bond takes place (**16'** to **16''**) followed by radical recombination to give *cis*-divinylcyclopropane (**9**). Pathway B proceeds through the formation of planar allylic anion **17**, which undergoes inversion to give *cis*-divinylcyclopropane (**9**). An alternative reaction pathway of the *trans*-divinylcyclopropane (**15**) to yield the cycloheptadiene product is the direct formation of the seven membered ring from diradical **16**.

### Biosynthetic applications

**Scheme 4:**
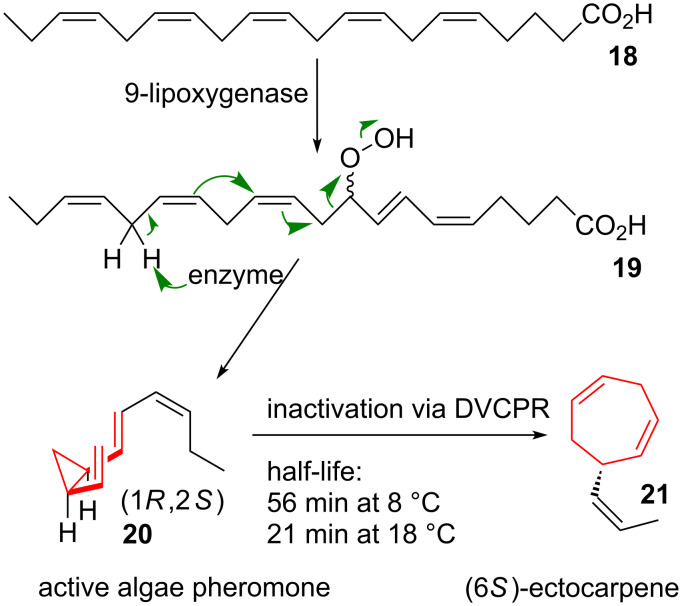
Proposed biosynthesic pathway to ectocarpene (**21**), an inactive degradation product of a sexual pheromone of brown algae (**20**).

The DVCPR has been shown to be part of the biosynthesis of ectocarpene (**21**, see Scheme 4) [29], an inactivated algae pheromone [30–31]. Starting from all-*cis*-pentaenic acid (**18**) peroxidation is supposed to take place to give **19**, followed by formation of the active algae pheromone **20** upon enzyme catalyzed fragmentation. Uncatalyzed DVCPR takes places to give **21** within short periods of time. The corresponding activation energies have been determined as well as the half-lifes at different temperatures. Bioassays proved that **20** was far more bioactive than **21**, and since the measured half-life for the conversion from **20** to **21** was longer than the necessary time for sexual encounter of the algae cycloheptadiene **21** was ruled out as a time-dependent sexual pheromone [32].

**Scheme 5:**
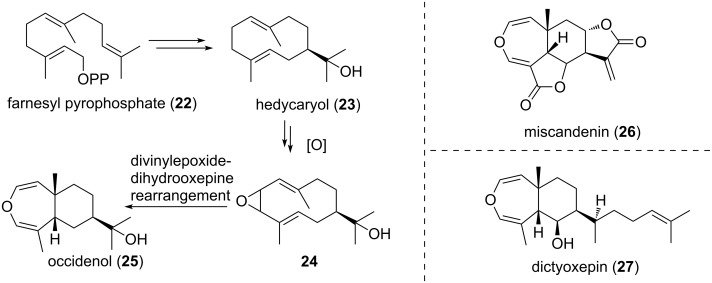
Proposed biosynthesis of occidenol (**25**) and related natural compounds.

A divinyloxirane rearrangement has been proposed as the key step in the biosynthesis of several natural products containing a dihydrooxepine moiety. In the case of occidenol (**25**, see Scheme 5), which has been isolated from the wood of *Thuja koraiensis,* farnesyl pyrophosphate (**22**) is supposed to undergo ring closure and the intermediate carbocation is trapped by hydroxide to give hedycaryol (**23**) [33]. Oxidation leads to divinyloxirane **24**, which rearranges to the corresponding dihydrooxepine, yielding occidenol (**25**) [34–35]. Miscandenin (**26,** isolated from *Mikania* species) [36] and dictyoxepin (**27**, isolated from brown algaes) [37] are supposed to originate from the same *cis*-divinyloxirane rearrangement.

**Scheme 6:**
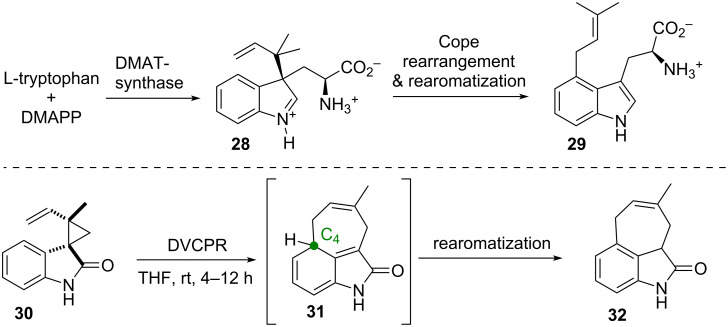
Gaich’s bioinspired system using the DVCPR to mimick the dimethylallyltryptophan synthase. DMAPP = dimethylallyl pyrophosphate.

Gaich et al. [38–39] used the DVCPR in a biosynthetic investigation targeting the dimethylallyltryptophan synthase. In order to test the biosynthetic hypothesis of the mode of action of the 4-prenylation of indoles by Arigoni and Wenkert (starting from L-tryptophan and dimethylallyl pyrophosphate (DMAPP) through **28** to yield **29**, (Scheme 6) [40–42] the spiro-oxindole **30** was synthesized. The system underwent a *cis*-aryl-vinyl-cyclopropane rearrangement [43] to give **31** followed by rearomatization in 4–12 hours at room temperature yielding tricycle **32**. The formation of the obtained tricyclic cyclohepta[*cd*]oxindole core **32** proved the synthetic versatility of a [3,3]-sigmatropic rearrangement for direct C–C-bond formation at the C4 position of the indole nucleus, and thus provides experimental evidence for the biosynthetic proposal.

### Applications to natural product synthesis

#### Fatty acid metabolites

**Scheme 7:**
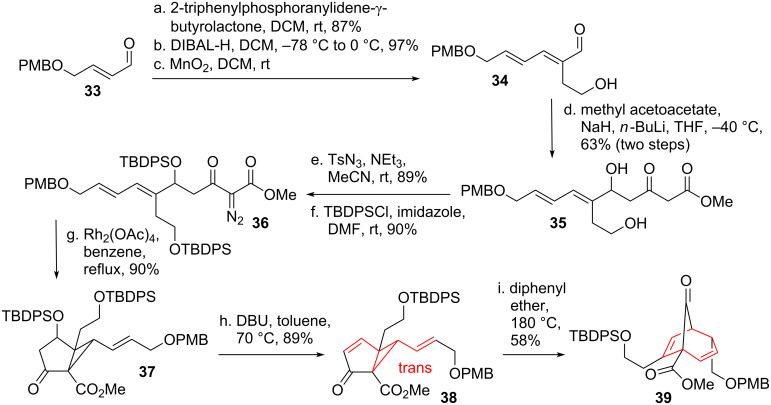
Iguchi’s total synthesis of clavubicyclone, part 1.

Iguchi and co-workers [44] applied the DVCPR to the total synthesis of the marine prostanoid clavubicyclone [45]. Known aldehyde **33** (see Scheme 7) [46] was subjected to Wittig conditions to furnish an intermediate lactone, which was then opened reductively followed by selective oxidation of the allylic alcohol to yield aldehyde **34**. Addition of double deprotonated methyl acetoacetate gave β-ketoester **35**. Diazotransfer followed by double protection resulted in the formation of compound **36**. Rh-catalyzed intramolecular cyclopropanation of this compound gave bicycle **37**. Selective removal of the secondary protected alcohol through β-elimination yielded *trans*-divinylcyclopropane **38**, which underwent DVCPR under forcing conditions after *trans*-*cis*-isomerization to obtain the desired bridged bicycle **39**.

**Scheme 8:**
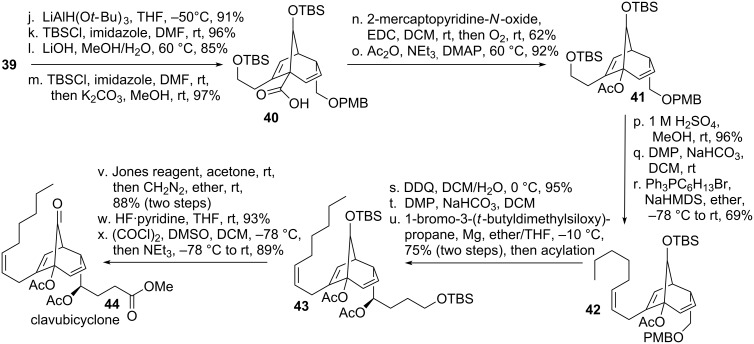
Iguchi’s total synthesis of clavubicyclone, part 2.

Reduction of ketone **39** (see Scheme 8) introduced a new stereocenter (10:1 selectivity), which was not assigned. The resulting major alcohol was protected, followed by saponification of the ester with concomitant removal of the TBDPS-protecting group. The resulting free alcohol was then re-protected to give bicycle **40**. Barton decarboxylation was then achieved using standard conditions, followed by trapping of the resulting carbon-centered radical with oxygen. The resulting tertiary alcohol was then protected to give acetate **41**. Standard functional group interconversions led to the formation of the remaining side chain using Wittig-conditions to afford olefin **42**. Another series of standard functional group interconversions furnished diacetate **43**, which could be converted to the desired natural product **44** using a three step sequence.

#### Terpenoid targets

**Scheme 9:**
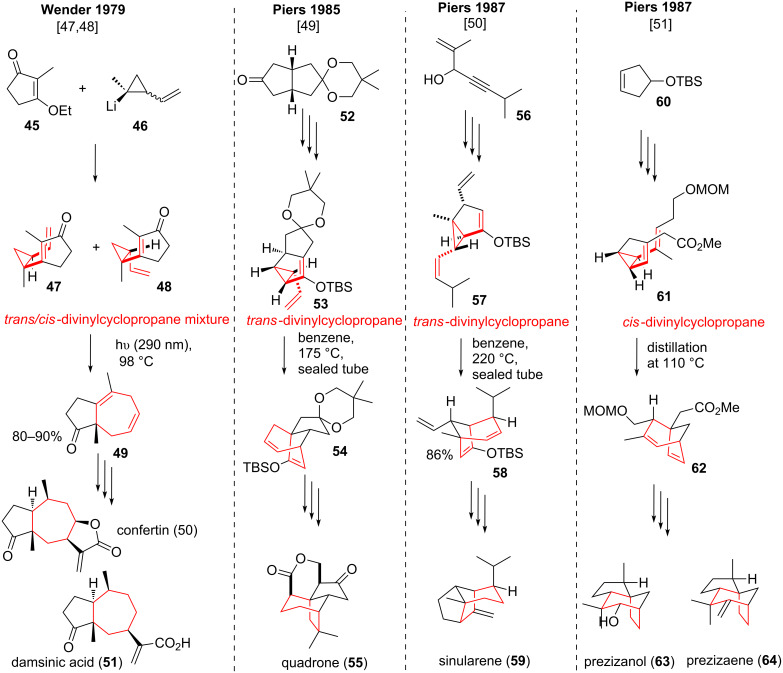
Wender’s syntheses of the two pseudoguainanes confertin (**50**) and damsinic acid (**51**) and Pier’s approaches towards four sesquiterpenoid natural products using the DVCPR with both *trans*- and *cis*-divinylcyclopropanes. The yields for **53** to **54** and **61** to **62** are stated as yield over several steps (77% over three steps and 98% over two steps, respectively).

The group of Wender applied the DVCPR in their total syntheses of (±)-confertin (**50**) and (±)-damsinic acid (**51**) [47]. A mixture a *trans*/*cis*-vinylcyclopropyllithium **46** (see Scheme 9) [48] was added to ketone **45** followed by elimination to give a mixture of *trans*/*cis*-divinylcyclopropanes **47**/**48**. Photoepimerization at the temperature required for *cis*-DVCPR (98 °C, *trans*-DVCPR occurred above 140 °C) gave bicyclic **49** in good yields. (±)-Confertin (**50**) and (±)-damsinic acid (**51**) could be accessed after 10 or 7 steps respectively.

Piers and co-workers were the first to examine the DVCPR as a key step in the formal synthesis of (±)-quadrone (**55**, see Scheme 9) [49], and the total syntheses of sinularene (**59**) [50], prezizanol (**63**) and prezizaene (**64**) [51]. The synthesis of the cytotoxic sesquiterpenoid quadrone (**55**) from *Aspergillus terreus* [52–53] started from tricyclic ketone **52** [54], which was converted into tricycle **53** in 12 steps. *Trans*-divinylcyclopropane **53** underwent the desired DVCPR after *trans*-*cis*-isomerization of the vinyl moiety at 175 °C, forming bridged tetracycle **54**, which already contained the crucial seven-membered ring present in the target molecule **55**. Intermediate **54** was advanced in ten steps to provide an intermediate aldehyde, which had been previously converted to the natural product in seven steps [55–57].

The total synthesis of the sesquiterpenoid sinularene (**59**) from the coral *Sinularia mayi* [58] started from alcohol **56**, which was converted to bicyclic **57** in nine steps. *Trans*-divinylcyclopropane **57** was then subjected to high temperature DVCPR conditions furnishing bridged bicycle **58**. *Trans*-*cis* isomerization of the vinyl moiety had to take place prior to the rearrangement to access the required boat transition state of the rearrangement. Intermediate **58** was converted to the natural product **59** in four additional steps.

Two other sesquiterpenoids, prezizaene (**63**) [59–60] and prezizanol (**64**) [60], isolated from *Eremophilia georgei* or vetiver oil were synthesized starting from cyclopentene derivative **60** [61]. This rather simple starting material was elaborated to advanced bicyclic **61** in only seven steps. This time the group of Piers used a *cis*-divinylcyclopropane, which underwent smooth DVCPR during distillation at 110 °C to yield bridged bicycle **62**. The desired natural products **63**/**64** were obtained after another ten or eleven steps respectively. For more “classic” applications of the DVCPR in total synthesis see reference [5].

**Scheme 10:**
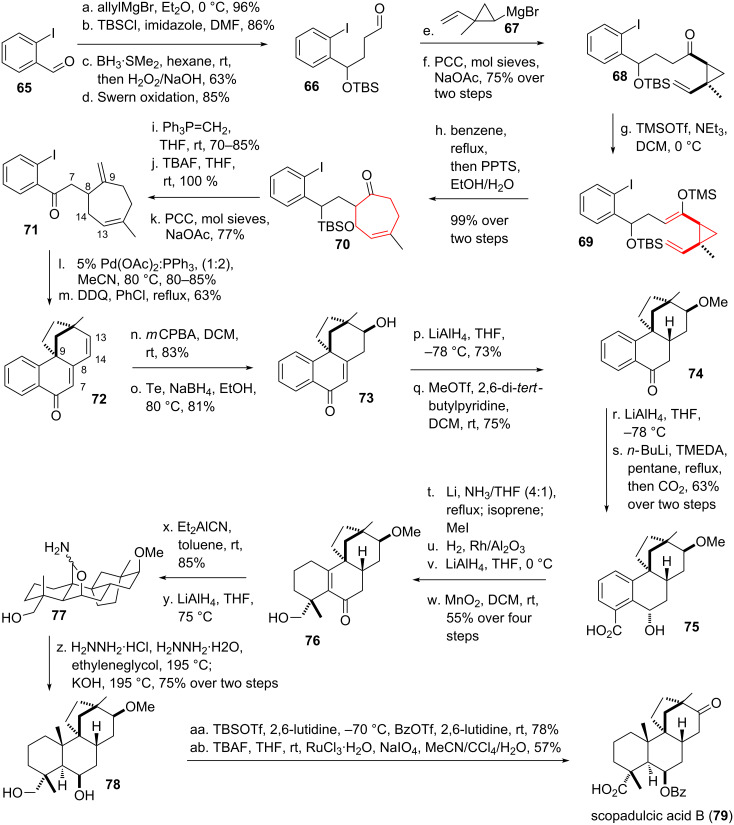
Overman’s total synthesis of scopadulcic acid B.

Overman and coworkers [62–65] successfully applied the DVCPR as the key step in their total synthesis of the diterpene scopadulcic acid B (**79**, see Scheme 10), isolated from the Paraguayan plant *Scoparia dulcis* [66]. Starting from 2-iodobenzaldehyde (**65**) allyl-Grignard addition took place followed by TBS-protection of the resulting alcohol. The installed double bond was subjected to hydroboration/oxidation, followed by Swern oxidation of the resulting alcohol to yield aldehyde **66**. Addition of cyclopropyl-Grignard reagent **67** [67–68], followed by oxidation to the corresponding ketone yielded vinylcyclopropane **68**. Deprotonation and TMS-protection furnished silyl-enolether **69**, which underwent the desired DVCPR in almost quantitative yield at elevated temperature, followed by removal of the silyl-protecting group under acidic conditions to furnish cycloheptenone **70**. Standard functional group interconversion furnished diolefin **71**. Subjection of this compound to Heck coupling conditions resulted in in the formation of a bridged tetracycle after initial Heck coupling followed by carbo-palladation and subsequent β-hydride elimination [69–71]. Double bond regioisomers (between C7 & C8 or C13 & C14) were obtained. Oxidation with DDQ yielded the 1,6-unsaturated ketone **72**. Selective epoxidation followed by reductive epoxide opening furnished the desired alcohol **73** [72]. Alcohol-directed 1,4-reduction using LiAlH_4_ [73] followed by ether formation gave methyl ether **74**. Reduction of the remaining ketone moiety gave the equatorial alcohol exclusively. Ortho-lithiation followed by the addition of carbon dioxide [74–75] resulted in the formation of carboxylic acid **75**. Birch reduction with concomitant methylation [76–77] followed by selective hydrogenation and reduction of the carboxylic acid resulted in the formation of alcohol **76**. Installation of the remaining quarternary carbon center was achieved by the conjugate addition of cyanide [78]. Reduction of the newly introduced nitrile yielded the unexpected stable pentacycle **77**. Formation of the missing methyl group (compound **78**) could be achieved using forcing Wolff–Kishner-conditions. Benzoate formation and global oxidation [79–80] finally furnished scopadulcic acid B (**79**). A similar approach was used by the same group in their total synthesis of scopadulcic acid A [81].

**Scheme 11:**
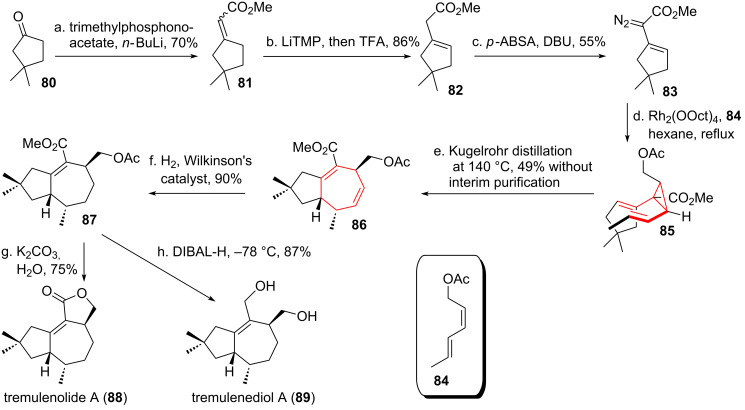
Davies’ total syntheses of tremulenolide A and tremulenediol A.

Davies and coworkers [82] utilized the DVCPR embedded in a formal [4 + 3]-cycloaddition [83] in the total syntheses of the related sesquiterpene metabolites tremulenolide A (**88**, see Scheme 11) and tremulenediol A (**89**), isolated from a fungal pathogen [84]. Horner–Wadsworth–Emmons olefination of the starting ketone **80** [85] provided an *E*/*Z*-mixture of α,β-unsaturated ester **81**. Deprotonation followed by an acidic quench resulted in deconjugation to give β,γ-unsaturated ester **82**. Diazotransfer using *p-*ABSA [86] yielded diazoester **83**. Selective rhodium-catalyzed cyclopropanation of the *cis*-double bond [87–88] of diene **84** [89] furnished *cis*-divinylcyclopropane **85**, which underwent DVCPR upon Kugelrohr distillation at 140 °C to give bicyclic **86**. Selective hydrogenation of the less substituted double bond using Wilkinson’s catalyst [90] gave α,β-unsaturated ester **87**. Removal of the acetate protecting group with concomitant lactonization concluded the total synthesis of tremulenolide A (**88**), whereas global reduction resulted in the formation of tremulenediol A (**89**).

**Scheme 12:**
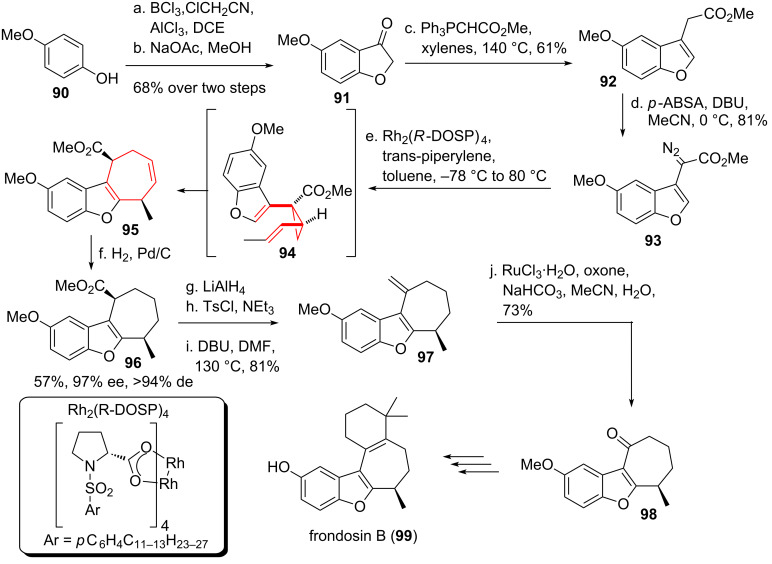
Davies formal [4 + 3] cycloaddition approach towards the formal synthesis of frondosin B.

Davies and co-worker [91–92] investigated the formal synthesis of the sequiterpene-hydroquinone derivative frondosin B (**99**, see Scheme 12) [93] via an enantioselective cyclopropanation of *trans*-piperylene and subsequent divinylcyclopropane rearrangement, to further demonstrate the versatility of their formal [4 + 3] cycloaddition [82]. Starting from 4-methoxyphenol (**90**) Friedel–Crafts acylation and cyclization provided bicycle **91**. Wittig olefination furnished benzofuran **92**. Diazotransfer using *p-*ABSA yielded the crucial diazo compound **93**, which was used in the following enantioselective cyclopropanation with the lesser substituted double bond of piperylene under Rh_2_(*R-*DOSP)_4_ catalysis. *Cis*-divinylcyclopropane intermediate **94** underwent in situ DVCPR under the reaction conditions, and rearomatization of the benzofuran moiety provided **95**. Reduction of the less hindered double bond yielded tricycle **96**, for which the yield and enantioselectivity was determined starting from **93**. Standard functional group interconversion first provided *exo*-methylene **97**, followed by ruthenium-catalyzed oxidative cleavage of the installed *exo*-methylene moiety [94] to give tricyclic ketone **98**, which can be converted to frondosin B (**99**) in three steps according to Danishefsky and co-workers [95–96].

**Scheme 13:**
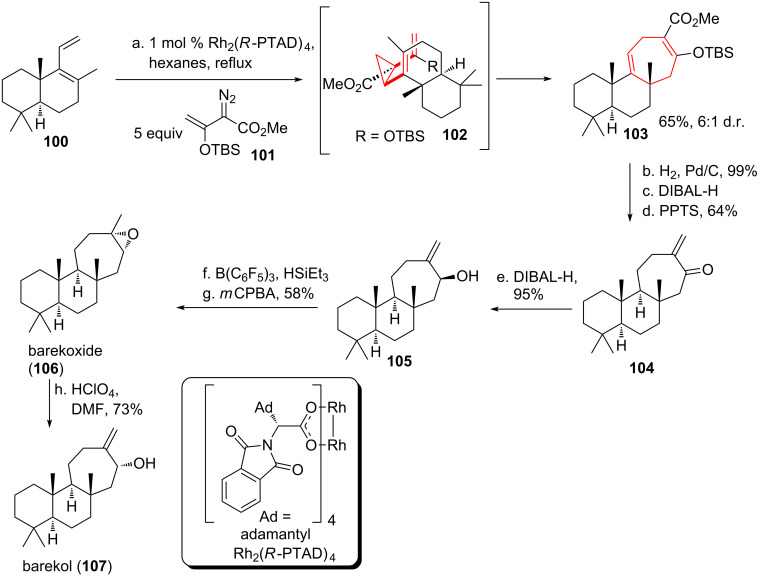
Davies and Sarpongs formal [4 + 3]-cycloaddition approach towards barekoxide (**106**) and barekol (**107**), involving a DVCPR.

The groups of Davies and Sarpong [97] teamed up for the total syntheses of the diterpenoids barekoxide (**106**, see Scheme 13) [98] and barekol (**107**) [99], isolated from the sponges *Chelonaplysilla erecta* and *Raspailia sp.* They envisioned a formal [4 + 3]-cycloaddition with an intermediate DVCPR [82]. Starting from diene **100,** enantioselective cyclopropanation catalyzed by Rh_2_(*R*-PTAD)_4_ took place using vinyldiazo compound **101** to form *cis*-dinvinylcyclopropane intermediate **102**. Rearrangement yielded tricycle **103** in both good yield and good diastereomeric ratio. Selective hydrogenation of the more electron rich double bond followed by reduction of the ester furnished α,β-unsaturated ketone **104** after PPTS-catalyzed elimination of water. Subsequent DIBAL-H reduction yielded the alcohol epimer **105** of barekol (**107**). Deoxygenation with concomitant isomerization of the double bond according to Gevorgyan [100] was followed by epoxidation to provide barekoxide (**106**). Acid-catalyzed isomerization finally yielded barekol (**107**).

**Scheme 14:**
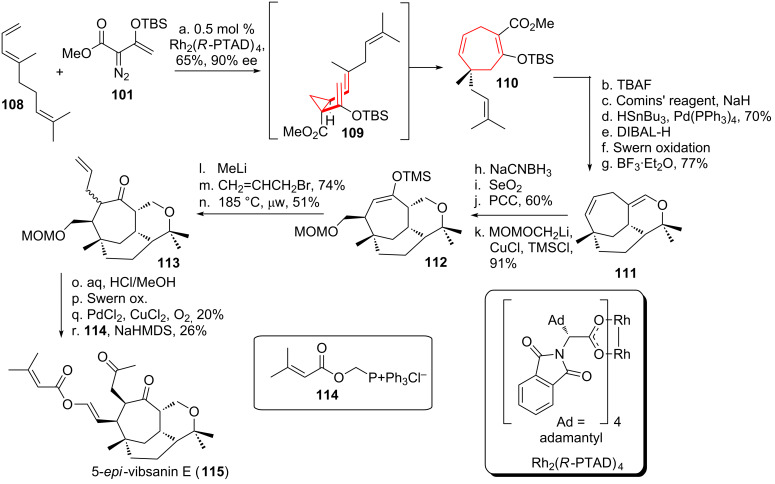
Davies formal [4 + 3]-cycloaddition approach to 5-*epi-*vibsanin E (**115**) containing an intermediate *cis*-divinylcyclopropane and the corresponding DVCPR.

Davies and coworkers [101–103] used the formal [4 + 3]-cycloaddition approach to access the diterpene 5-*epi*-vibsanin E (**115**), from the plant *Viburnum awabuki* (see Scheme 14) [104]. Starting from triene **108** cyclopropanation was achieved using vinyldiazo compound **101**. The fomal [4 + 3]-cycloaddition proceeded through *cis*-divinylcyclopropane **109** to yield rearranged cycloheptadiene **110**. Desilylation was achieved using TBAF, followed by formation of a vinyl triflate and Stille coupling with tributyltin hydride. The oxidation state of the remaining ester was adjusted to the corresponding aldehyde, followed by a Lewis acid-catalyzed intramolecular inverse-electron-demand hetero-Diels–Alder reaction to give tricycle **111**. The enol ether moiety was reduced using NaCNBH_3_, followed by allylic Riley oxidation and PCC-mediated enone formation. Copper-catalyzed conjugate addition in the presence of TMSCl [105] yielded silyl enol ether **112**. Subsequent introduction of the side chain in **113** via a Claisen rearrangement was followed by MOM-cleavage. Oxidation furnished an intermediate aldehyde, followed by Wacker oxidation and the Anders–Gaßner variant [106–109] of the Wittig reaction to furnish 5-*epi*-vibsanin E (**115**).

**Scheme 15:**
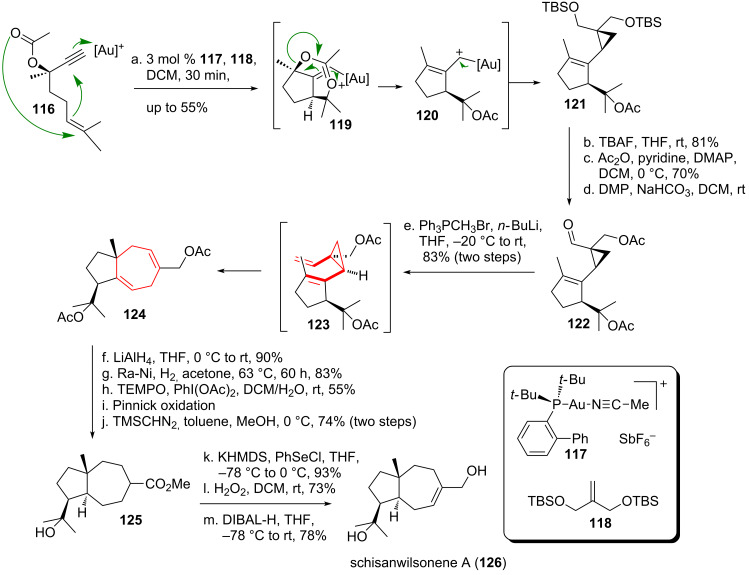
Echavarren’s total synthesis of schisanwilsonene A (**126**) featuring an impressive gold-catalzed cascade reaction and a DVCPR.

Echavarren and coworkers [110] used a DVCPR to target the sesquiterpenoid schisanwilsonene A (**126**, see Scheme 15), isolated from *Schisandra wilsoniana* [111], a plant used in traditional chinese medicine. Submission of 1,6-enyne **116** to cationic gold-catalyst **117** led to 5-*exo*-*dig* cyclization and intermediate formation of bridged bicycle **119**. Subsequent 1,5-acyl-shift afforded vinylcarbenoid **120**, which underwent cyclopropanation with the added olefin **118** to give bicycle **121** in decent yield. Double deprotection followed by selective acetal-protection of the less hindered alcohol and oxidation of the remaining unprotected alcohol moiety led to aldehyde **122**. Wittig olefination resulted in the formation of non-isolated *cis*-divinylcyclopropane **123**, which immediately underwent DVCPR to give **124** at ambient temperature. Ester **125** was obtained using standard functional group interconversions. The desired natural product schisanwilsonene A (**126**) was obtained after selective formation of the less hindered α,β-unsaturated ester and subsequent reduction.

#### Alkaloid targets

**Scheme 16:**
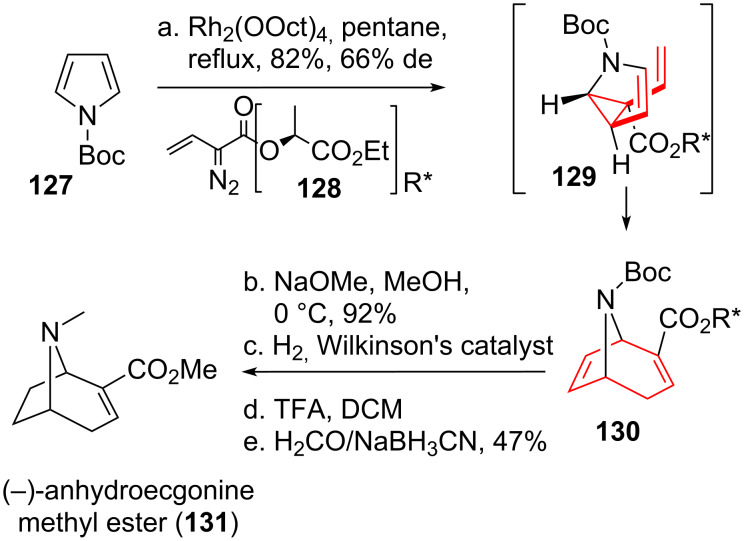
Davies early example of a formal [4 + 3]-cycloaddition in alkaloids synthesis.

Davies and co-worker [112] were the first to apply the DVCPR to the total synthesis of alkaloids. Anhydroecgonine methyl ester (**131**, see Scheme 16) is a tropane alkaloid structurally related to cocaine, which can be detected in the human body after cocaine consumption. It can be degradatively accessed from cocaine through pyrolysis, cocaine congeners can be prepared via conjugate addition afterwards [113–114]. Boc-protected pyrrole **127** was subjected to rhodium-catalyzed cyclopropanation with vinyldiazo compound **128** [115–116], bearing a chiral auxiliary [117]. The intermediate *cis*-divinylcyclopropane **129** rearranged to the corresponding bridged cycloheptadiene **130** in a DVCPR. The chiral auxiliary was removed in the following using methanolysis conditions, followed by reduction of the more electron rich double bond and reductive amination after Boc-deprotection to yield the desired natural product **131** in only five steps.

**Scheme 17:**
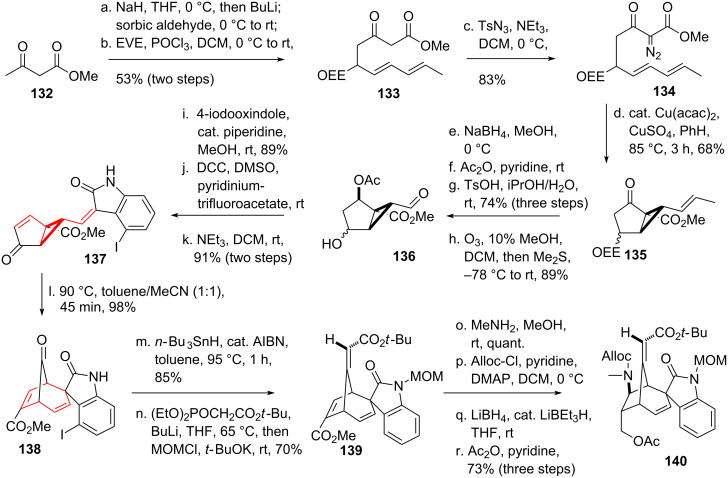
Fukuyama’s total synthesis of gelsemine, part 1.

**Scheme 18:**
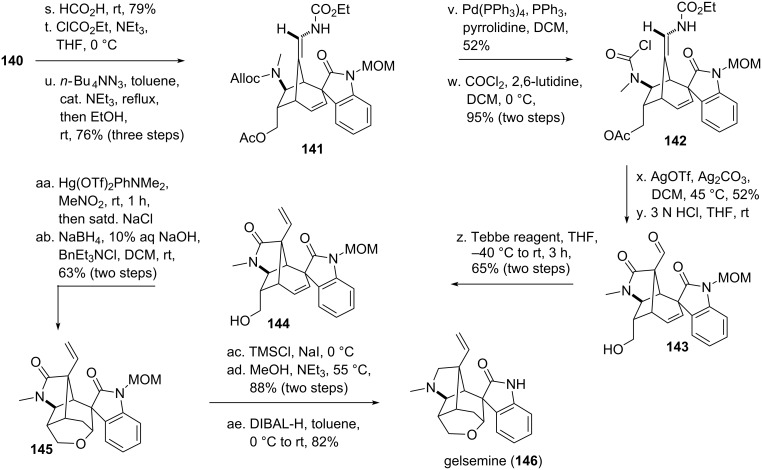
Fukuyama’s total synthesis of gelsemine, featuring a divinylcyclopropane rearrangement, part 2.

Perhaps one of the most prominent alkaloid syntheses using a DVCPR was carried out by Fukuyama and coworker [118–120] and targeted gelsemine (**146**, see Scheme 17 and Scheme 18), an alkaloid with a unique hexacyclic cage structure isolated from *Gelsemium Sempervirens* [121–122]. Starting from methyl acetoacetate (**132**), double deprotonation and addition of the more reactive anion to sorbal aldehyde furnished the corresponding alcohol, which was immediately protected as its acetal **133** using ethyl vinyl ether. The necessary diazo moiety was installed using tosylazide as a diazo-transfer reagent to yield **134**. Copper catalyzed intramolecular cyclopropanation furnished bicycle **135**. Reduction of the carbonyl moiety and protection of the resulting alcohol was followed by ether cleavage and ozonolysis to give aldehyde **136** [123]. Knoevenagel condensation with 4-iodooxindole was achieved in the next step. Pfitzner–Moffatt oxidation [124] followed by elimination furnished *trans*-divinylcycloproane **137**. Submission of this compound to elevated temperature initiated a very smooth DVCPR yielding bicycle **138**. The temperature for the predecessing *trans*-*cis* divinylcyclopropane isomerization was unusually low. This can be rationalized by the substitution of the divinylcyclopropane with two electron withdrawing groups and the resulting stabilization of the intermediate(s). The surplus iodine at the oxindole was crucial for the stereochemical control of the spiro-indolinone system in **138**, it was removed in the next step using radical conditions. Horner–Wadsworth–Emmons olefination on the carbonyl moiety followed by protection of the oxindole gave intermediate **139**. Conjugate addition of methylamine and protection of the amine was followed by selective reduction of the methyl ester [125]. Protection of the resulting alcohol gave tetracycle **140**.

The remaining ester **140** (see Scheme 18) was then converted to the corresponding acid, followed by anhydride formation. Introduction of an acyl azide resulted in a Curtius rearrangement, upon heating the intermediate isocyanate was trapped with EtOH to yield ethyl carbamate protected amine **141**. Deprotection of the Alloc-group [126] and chloro carbamate formation furnished **142**. Exposure of this compound to a silver source led to the formation of the five-membered lactam, together with an acylimine. This was removed under acidic conditions to furnish aldehyde **143**. Tebbe olefination [127] installed the missing double bond in **144**. Oxymercuration [128] followed by reductive biphasic demercurization [129] furnished the remaining tetrahydropyran ring, yielding **145**. Removal of the MOM-protecting group followed by reduction of the amide concluded Fukuyama’s total synthesis of gelsemine (**146**).

**Scheme 19:**
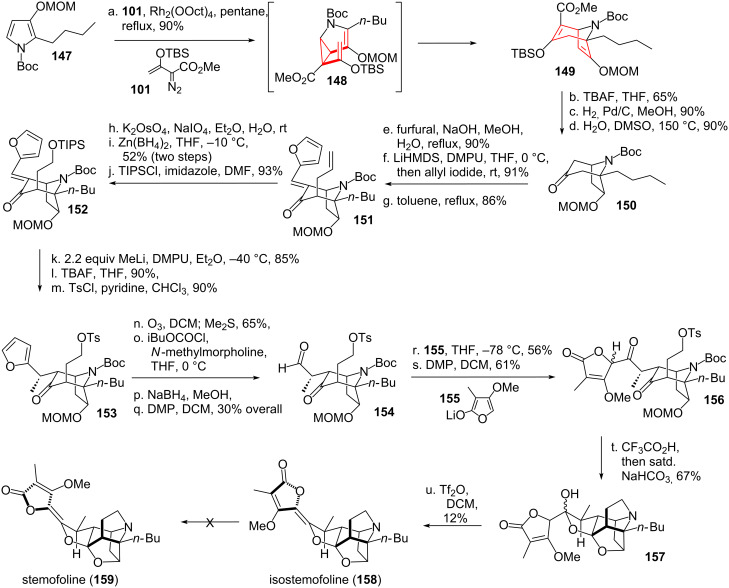
Kende’s total synthesis of isostemofoline, using a formal [4 + 3]-cycloaddition, including an intermediate DVCPR.

An approach similar to Davies formal [4 + 3]-cycloaddition [81] was used by the group of Kende [130] to access the alkaloid isostemofoline (**158**, see Scheme 19) [131]. Rhodium-catalyzed cyclopropanation of silyl-enol-diazo compound **101** and Boc-protected pyrrole **147** resulted in the formation of intermediate *cis*-divinylcyclopropane **148**, that rearranged under the reaction conditions to give the corresponding highly substituted bridged cycloheptadiene **149**. Deprotection of the enolate, reduction of the remaining double bond and subsequent Krapcho decarboxylation [132] resulted in less functionalized ketone **150**. Aldol condensation with furfural followed by *O*-allylation and Claisen rearrangement furnished enone **151**. Standard functional group interconversiones were used to access TIPS-protected alcohol **152**. Addition of methyllithium at low temperature [133] resulted in stereoselective conjugate attachment of the required methyl group. Deprotection of the alcohol and transformation into a suitable leaving group yielded tosylate **153**. Next, the furan was cleaved oxidatively, the resulting acid was converted to the corresponding anhydride, which could be reduced to the alcohol using NaBH_4_, followed by reoxidation with DMP to yield aldehyde **154**. Attachment of furanone enolate **155** [134–135], followed by reoxidation yielded tricycle **156**. Deprotection of the amine and the MOM-protected alcohol led to a rearrangement cascade, which smoothly yielded hexacycle **157**. This compound could be converted into isostemofoline (**158**), albeit in low yield. Isostemofoline could not be interconverted into stemofoline (**159**) using trifluoroacetic acid [136].

**Scheme 20:**
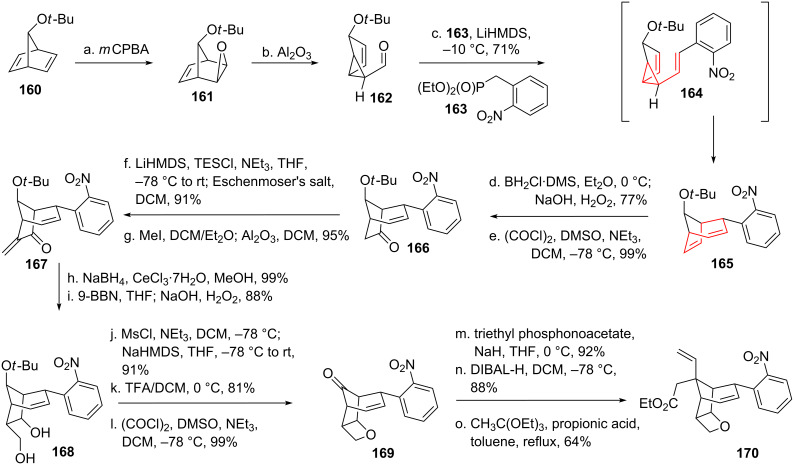
Danishefsky’s total synthesis of gelsemine, part 1.

**Scheme 21:**
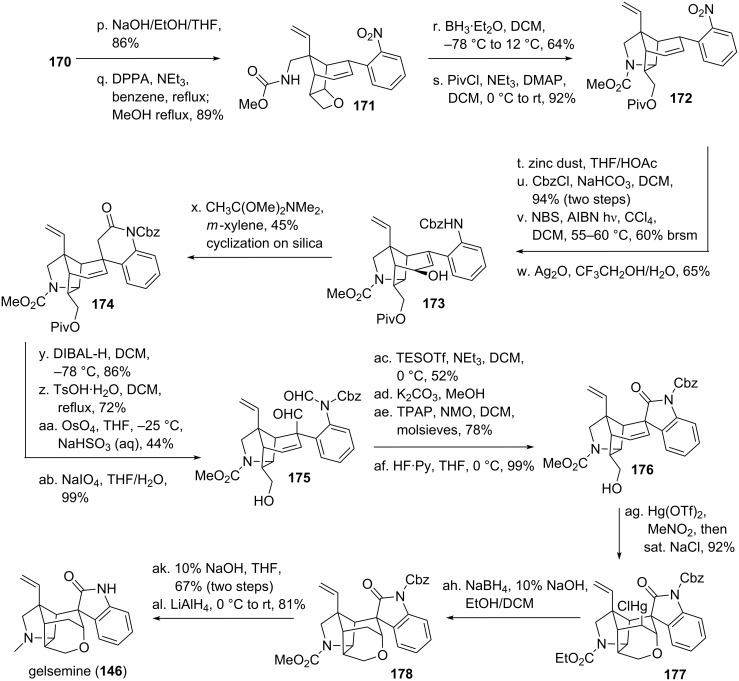
Danishefsky’s total synthesis of gelsemine, part 2.

Danishefsky and coworkers [137–139] applied the DVCPR in their total synthesis of gelsemine (**146**, see Scheme 20 and Scheme 21). Starting from bicycle **160** [140] epoxidation using *m*CPBA furnished epoxide **161** [141–142], which could be converted into vinylcyclopropanecarbaldehyde **162** upon rearrangement. Olefination using HWE-reagent **163** yielded intermediate *cis*-divinylcyclopropane **164**, which underwent the desired rearrangement at remarkably low temperature to yield bicycle **165**. Selective hydroboration/oxidation (directed through participation of the untouched double bond) followed by Swern oxidation gave ketone **166**. The enone system **167** was prepared through addition of Eschenmoser’s salt, followed by Hoffman elimination of the resulting amine. Luche reduction [143] from the less hindered side followed by hydroboration/oxidation gave diol **168**. Mono-mesylation of the primary alcohol followed by the addition of base furnished the signature oxetane moiety. Ether cleavage [144] and Swern oxidation resulted in the formation of ketone **169**. HWE-olefination followed by reduction to the allyl alcohol led to allylic ester **170** after a Johnson–Claisen rearrangement [145] upon treatment with triethyl orthoacetate.

Ester **170** was saponificated (see Scheme 21), followed by the formation of an acid-azide. Shiori version [146–147] of the Curtius rearrangement with concomitant addition of MeOH to the intermediate isocyanate afforded carbamate **171**. Oxetane **171** was then opened under Lewis-acidic conditions. The deprotected alcohol was protected to give pentacycle **172**. Reduction of the nitro group was followed by Cbz-protection. Allylic alcohol **173** resulted from radical allylic bromination followed by displacement of bromine through water under silver-catalysis. Eschenmoser–Claisen rearrangement [148] led to the formation of the remaining quarternary carbon center, the resulting amide cyclized during the purification on silica to give amide **174**. Reduction of this amide to the aminal was followed by dehydration and Lemieux–Johnson [149] oxidative cleavage to give dialdehyde **175**. The liberated alcohol moiety was reprotected, followed by cleavage of the amide under basic conditions. Cyclization and Ley–Griffith oxidation [150] took place, followed by deprotection of the alcohol to give spiro-oxindole **176**. The formation of the remaining tetrahydropyran was much in line with Fukuyama’s synthesis, utilizing the same oxymercuration/reductive demercurization [128–129] sequence (through intermediate **177**) yielding **178**. The oxindole protecting group was then removed, followed by reduction of the carbamate to the remaining missing methyl group [151] to finish gelsemine (**146**).

**Scheme 22:**
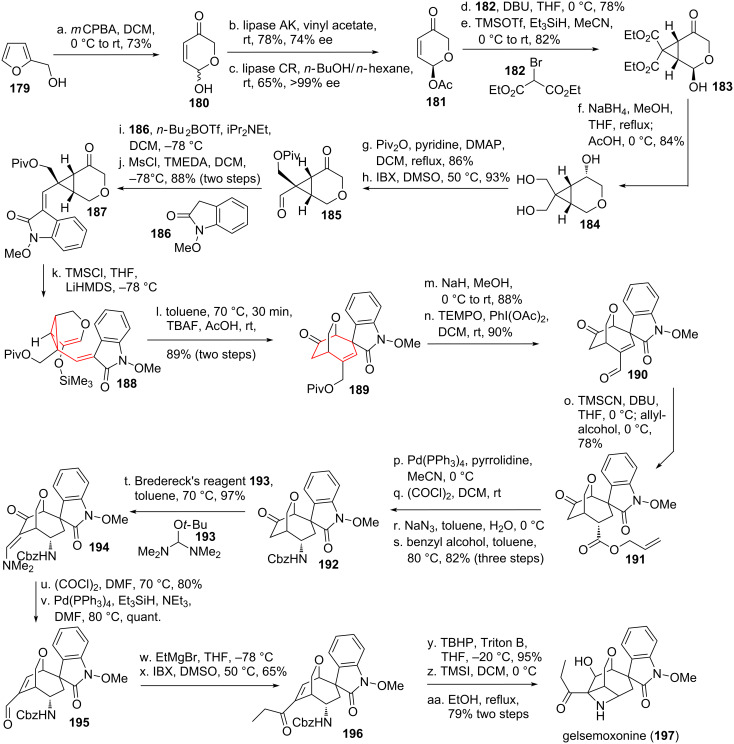
Fukuyama’s total synthesis of gelsemoxonine.

The total synthesis of the monoterpenoid-indole alkaloid gelsemoxonine (**197**, see Scheme 22) [152], isolated from the leaves of *Gelsemium elegans* was accomplished by Fukuyama and coworkers [153–154]. Starting from furfuryl alcohol (**179**) an epoxide initiated Achmatowicz reaction [155] took place to give α,β-unsaturated pyrane **180**. Next in line was an enzyme catalyzed dynamic kinetic resolution [156], albeit with unsatisfactory enantiomeric excess. The undesired enantiomer was then selectively cleaved using another enzyme with reversed selectivity to give enantiopure pyranone **181**. Cyclopropanation was achieved using a Michael addition initiated ring closure yielding diester **183**. Complete reduction furnished triol **184**, followed by mono-protection of the least hindered alcohol. The remaining alcohol moieties were then oxidized to the corresponding keto-aldehyde **185**. A two-step procedure was employed to generate α,β-unsaturated oxindole-*N*-methoxide **187** [157–158]. Formation of silyl enol ether **188** furnished the desired *cis*-divinylcyclopropane, which underwent smooth DVCPR under mild conditions to give bridged bicycle **189**. The alcohol was deprotected and oxidized to aldehyde **190**. The aldehyde was transferred into the corresponding cyanohydrin trimethylsilyl ether using TMSCN [159–161], followed by protonation of the TMS-enolate and esterification of the intermediate acyl cyanide yielding allyl ester **191**. This ester was transferred into the corresponding carboxylic acid, followed by formation of the acid chloride. The chloride was displaced with an azide, which underwent Curtius rearrangement upon heating. The intermediate isocyanate was intercepted by benzyl alcohol to provide secondary Cbz-protected amine **192**. Bredereck’s reagent (**193**) [162] was used to generate enamine **194**. The enol-form of the remaining carbonyl moiety was transformed into the corresponding vinyl chloride using Vielsmeier’s reagent [163–164]. Dechlorination [165–166] was then achieved and yielded α,β-unsaturated aldehyde **195** upon hydrolysis. Grignard addition to the newly formed aldehyde followed by reoxidation furnished the side chain of compound **196**. Treatment of unsaturated ketone **196** with Triton B and TBHP yielded the epoxide resulting from top-face attack. The surplus Cbz-group was then deprotected using TMSI [167–168]. The final azetidine formation took place upon refluxing in ethanol to give gelsemoxonine (**197**).

### Further synthetic applications

**Scheme 23:**
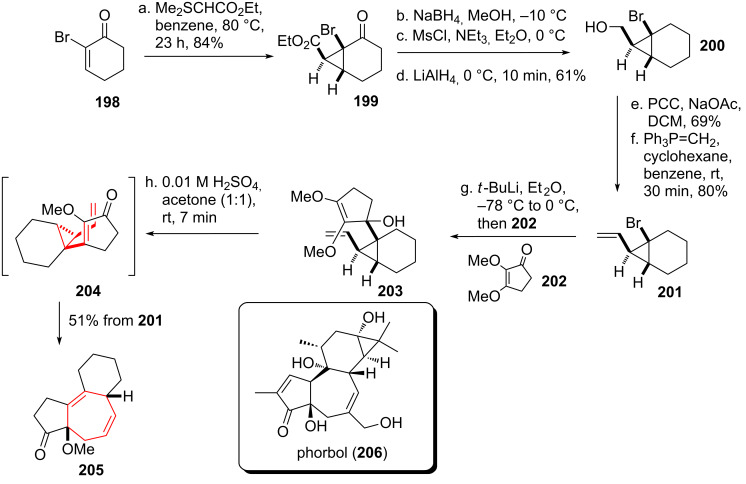
Wender’s synthetic access to the core skeleton of tiglianes, daphnanes and ingenanes.

The group of Wender applied the DVCPR in an approach towards the core skeleton of tiglianes (like phorbol **206**, see Scheme 23), daphnanes and ingenanes [169] in 1980 [170]. Starting from α-bromoenone **198** a Corey–Chaykovsky cyclopropanation reaction was achieved to yield cyclopropane **199**. The keto-group was removed using a three step sequence, with concomitant reduction of the ester to give alcohol **200**. Oxidation and Wittig olefination gave olefin **201**. Lithium bromide exchange followed by 1,2-addition to ketone **202** yielded tricyclic **203**, which was immediately subjected to acidic hydrolysis to give the desired tricyclic core skeleton **205** in 51% yield through transition state **204**.

**Scheme 24:**
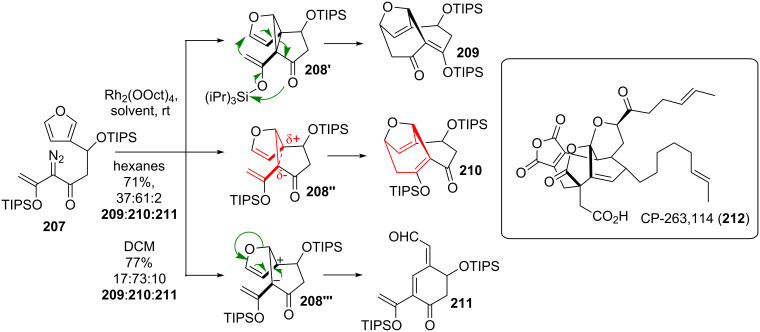
Davies’ approach towards the core skeleton of CP-263,114 (**212**).

The group of Davies [171] demonstrated the impressive synthetic power of the DVCPR in their core structure synthesis of CP-263,114 (**212**, see Scheme 24) [172–173]. Subjection of diazofuran **207** to rhodium catalysis resulted in the formation of **209**, **210** and **211** in solvent-dependent ratios. The sole product arising from the desired DVCPR via **208''** is the major product **210**. The two remaining products arise from *trans*-silylation and fragmentation (**208'** to **209**) or via ionic intermediate **208'''** and fragmentation to give **211**. The different product distribution can be controlled by the choice of the solvent. Notably cycloheptadiene **210** arising from the DVCPR contains two as well as the related *trans*-silylated compound **209**. This demonstrates the huge driving force of the DVCPR, as two highly strained olefins are favoured over a cyclopropane-moiety.

**Scheme 25:**
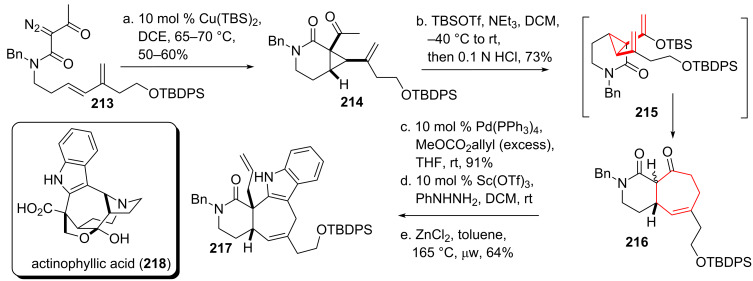
Wood’s approach towards actinophyllic acid.

Wood and coworkers [174] applied the DVCPR in the core structure synthesis of actinophyllic acid (**218**, see Scheme 25) [175]. Subjection of diazo compound **213** to copper catalysis yielded vinylcyclopropane **214**. Deprotonation and silyl ether formation resulted in intermediate *cis*-divinylcyclopropane **215**, which smoothly underwent DVCPR to give cyclohexane **216**. Tsuji–Trost allylation [176–177] furnished the quartenary carbon center. A two-step Fischer-indole strategy [178–179] finished tetracycle **217** under forcing conditions.

**Scheme 26:**
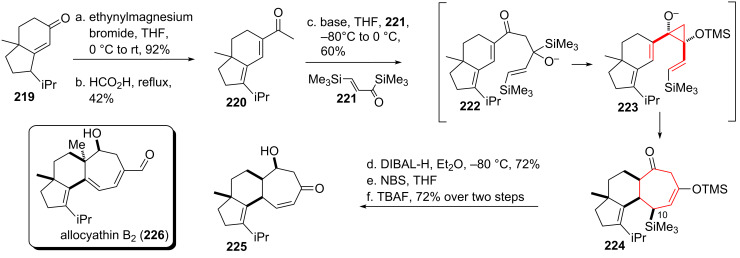
Takeda’s approach towards the skeleton of the cyanthins, utilitizing the divinylcyclopropane rearrangement in a [4 + 3] cycloaddition.

Takeda and coworkers [180] set out to investigate the use of an anionic oxy-*cis*-divinylcyclopropane rearrangement to build up the diterpenoid cyanthin skeleton [181–183] like that of allocyathin B_2_ (**226**, see Scheme 26), which was isolated from bird’s nest fungi [184–189]. Starting from α,β-unsaturated ketone **219** addition of ethynylmagnesium bromide took place, followed by a Rupe rearrangement [190] using refluxing acetic acid as solvent to give ketone **220**. Addition of deprotonated ketone **220** onto acryloylsilane **221** [191] gave alkoxy intermediate **222**, which underwent a Brook rearrangement followed by cyclopropane formation to yield anionic **223** in situ [192]. The aforementioned anionic oxy-*cis*-divinylcyclopropane rearrangement took place, yielding tricycle **224**. DIBAL-H reduction gave the corresponding alcohol stereoselectivly, followed by removal of the TMS-group at C10 with NBS and further enone formation upon treatment with TBAF to give tricyclic core skeleton **225** [192]. This reaction sequence constitutes a very nice example of a formal [4 + 3]-cycloaddition, without the use of a transition metal catalyst.

**Scheme 27:**
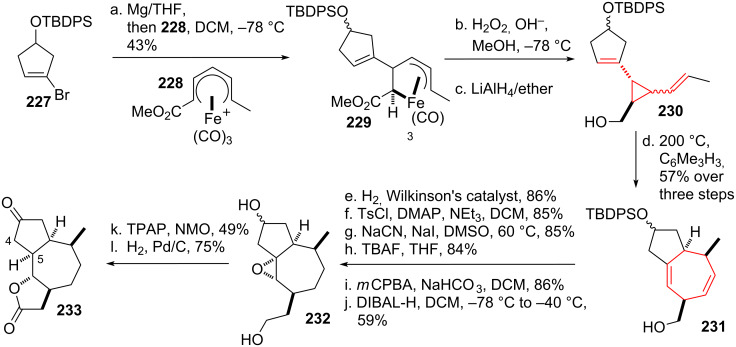
Donaldson’s organoiron route towards the guianolide skeleton.

Donaldson and coworkers [193] used the DVCPR en route towards the core skeleton of the sesquiterpenoid guianolide family [194–195]. Starting from readily prepared vinyl bromide **227** (see Scheme 27) formation of the corresponding Grignard species was acomplished, followed by addition to organoiron complex **228** to give (pentenediyl)iron complex **229**. Oxidation led to the formation of the desired divinylcyclopropane, followed by reduction of the ester to the desired alcohol to give compound **230**. Subjection of the *trans*-*cis*-divinylcyclopropane mixture to elevated temperature smoothly formed the desired bicycle **231**. The less hindered double bond was removed using Wilkinson’s catalyst, followed by standard functional group interconversions to yield epoxide **232**. When this compound was subjected to oxidative conditions both alcohols were oxidized to the corresponding aldehyde/ketone. Base-induced epoxide opening led to the formation of a double bond (C4/C5) and concomitant lactol-formation. The lactol was oxidized to the corresponding lactone under the same reaction condition. Final reduction gave core skeleton **233**.

#### DVCPR in tandem reactions

**Scheme 28:**
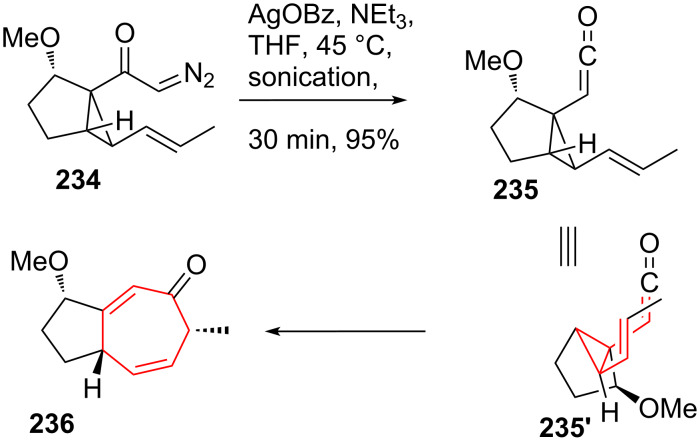
Stoltz’s tandem Wolff/DVCPR rearrangement.

The group of Stoltz [196–197] succeeded in establishing a tandem Wolff rearrangement/divinylcyclopropane rearrangement strategy [198]. Readily accessible α-diazo ketone **234** (see Scheme 28) was shown to undergo Wolff rearrangement [199] upon treatment with silver benzoate. The intermediate ketene **235** underwent stereospecific DVCPR through transition state **235'** under the reaction conditions to give enone **236** in excellent yield. This constituted the first example of the direct formation of an enone through a DVCPR.

**Scheme 29:**
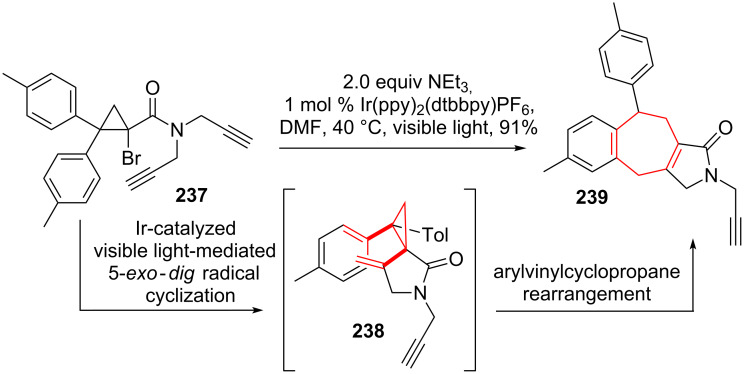
Stephenson’s tandem photocatalysis/arylvinylcyclopropane rearrangement.

Stephenson and coworker [200] found an intriguing example of a light mediated radical cyclization/arylvinylcyclopropane rearrangement. Subjecting cyclopropyl bromide **237** to an Ir-polypyridyl catalyst and visible light initiated the desired photoredox cascade forming a cyclopropylradical, which readily cyclized in an 5-*exo*-*dig* fashion. Radical quenching gave *cis*-arylvinylcyclopropane **238**. Arylvinylcyclopropane rearrangement was followed by rearomatization to give tetracycle **239** (see Scheme 29).

**Scheme 30:**
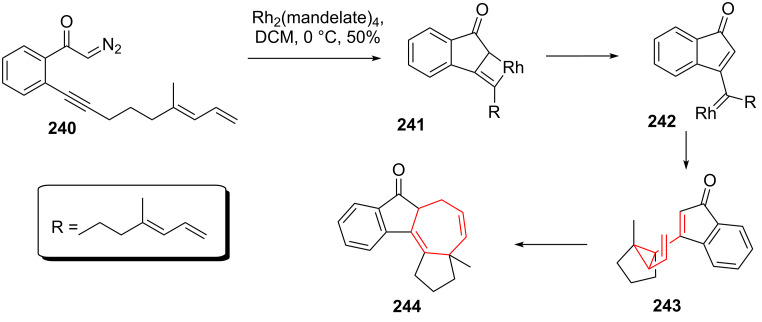
Padwa’s rhodium cascade involving a DVCPR.

Padwa and coworkers [201] discovered an intermediate DVCPR during their investigation of rhodium-catalyzed cyclizations of alkynyl substituted α-diazo ketones. Transition metal-catalyzed diazo-decomposition of compound **240** (see Scheme 30) resulted in the formation of metallacyclobutene **241**, followed by rapid metallacycloreversion to give carbenoid species **242**. Intramolecular cyclopropanation furnished divinylcyclopropane **243**, which underwent DVCPR under these conditions to give tetracycle **244** in 50% yield.

**Scheme 31:**
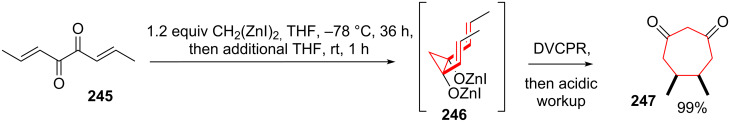
Matsubara’s version of a DVCPR.

Matsubara and coworkers [202] investigated the formation of cyclohepta-1,3-diones from 1,2-diketone starting materials. Treatment of **245** with *bis*(iodozincio)methane resulted in the formation of *cis*-divinylcyclopropane **246** as the corresponding *bis*-zinc-enolate species. DVCPR occurred at ambient temperature, final acidic workup provided cycloheptadione **247 i**n excellent yield (see Scheme 31).

**Scheme 32:**
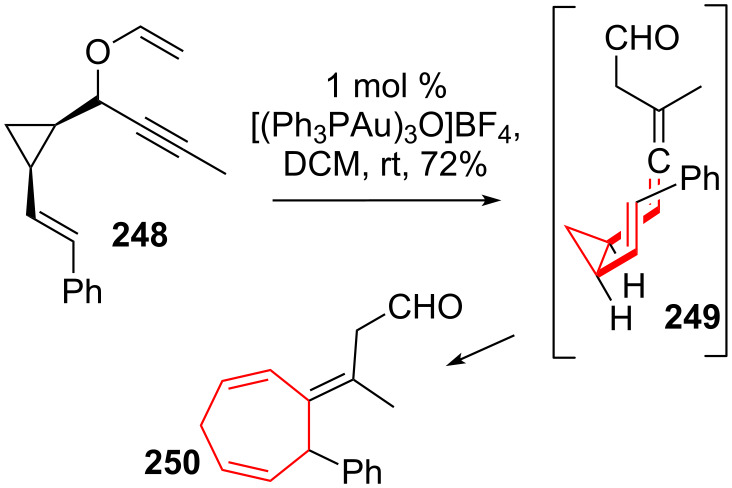
Toste’s tandem gold-catalyzed Claisen-rearrangement/DVCPR.

Toste and coworkers [203] reported a tandem gold-catalyzed Claisen rearrangement from popargyl vinyl ether **248** (see Scheme 32) to give intermediate vinyl-allenecyclopropane **249**, followed by a DVCPR to furnish cycloheptadiene **250**.

#### 1,2-Shift and vinyl-carbenoid formation sequences

**Scheme 33:**
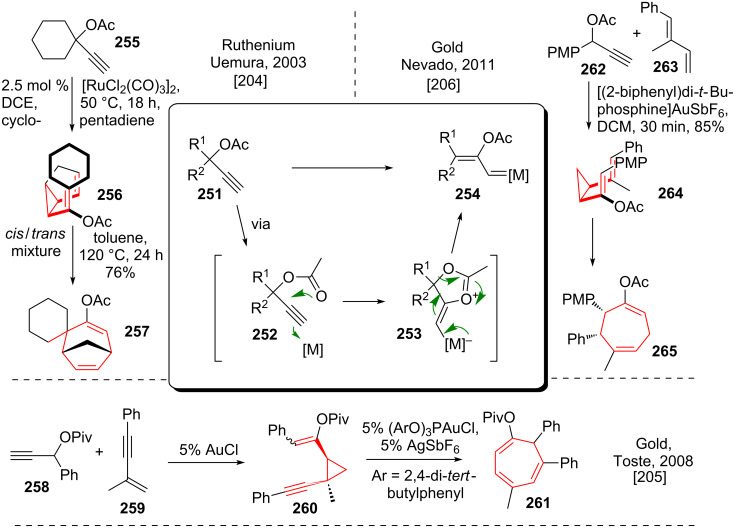
Ruthenium- and gold-catalyzed versions of tandem reactions involving a DVCPR.

Two major pathways to generate divinylcyclopropanes using transition metal catalysis have been developed. Uemura and coworkers [204] were the first to apply the transition metal catalyzed 1,2-acyl shift with subsequent vinyl carbenoid formation. Propargylic acetate **251** (see Scheme 33) has been shown to undergo 5-*exo*-*dig* cyclization via **252** to give zwitterionic intermediate **253.** A concomitant fragmentation reaction yielded vinyl-carbenoid **254**. Uemura and coworkers used this chemistry with propargylic acetate **255** to achieve cyclopropanation with various dienes, for example cyclopentadiene, to generate a *cis*/*trans*-mixture of divinylcyclopropanes **256**. Heating of this mixture of compounds resulted in the formation of bridged tricycle **257** in good yield. Toste and coworkers [205] discovered a 1,2-pivaloyl shift and cyclopropanation of the resulting gold-carbenoid from **258** with enyne **259** to yield vinylalkynecyclopropane **260**. This compound was shown to undergo a gold catalyzed DVCPR to yield **261**. Note that this reaction does most likely proceed via a step wise mechanism and involves charged intermediates.

Nevado and coworkers [206] applied a closely related reaction to propargylic acetate **262** using a cationic gold(I) catalyst, which was used to selectively cyclopropanate the less hindered double bond of dienes like **263**. Spontaneous DVCPR provided cycloheptadiene **265** via **264** in good yields.

Further contributions to the topic have been put forward by the group of Echavarren [207] and Gung [208].

#### Cycloisomerization involving DVCPR

**Scheme 34:**
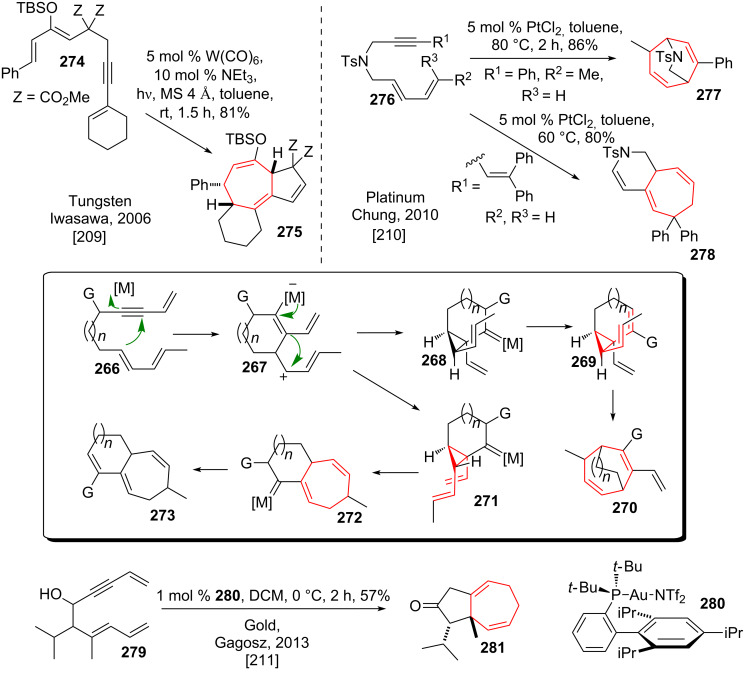
Tungsten, platinum and gold catalysed cycloisomerizations leading to a DVCPR.

Iwasawa and coworkers [209] discovered a cyclopropanation/DVCPR sequence of alkyne-substituted silyl enol ethers (for example **274**, see Scheme 34) catalyzed by in situ formed W(CO)_5_(tol) upon irradiation to give annulated tricycle **275**. The common mechanism for this type of reaction proceeds via *endo*-*dig* cyclization of enynes like **266** to give zwitterionic intermediate **267**. Metal-carbenoid formation with subsequent cyclopropane formation gives rise to either *cis*- or *trans*-divinylcyclopropanes **268**/**271**. *cis*-Divinylcyclopropane **271** can readily undergo DVCPR to give bicycle **272**. 1,2-Migration of the migrating group G leads to final bicycle **273** after regeneration of the catalyst. *Trans*-divinylcyclopropane **268** can undergo DVCPR after 1,2-migration and subsequent formation of *cis*-divinylcyclopropane **269** yielding bridged bicycle **270**.

Chung and coworkers [210] discovered a related reaction pattern using platinum(II) as the catalyst. Depending on the attached rests on enyne **276** both possible cycloheptadienes (bridged **277** or annulated **278**) could be accessed. The selective formation of annulated bicycle **278** in preference of the possible bridged variant underlined the prefered reactivity of their enyne system.

Gagosz and coworkers [211] recently showed that the cycloisomerization of enynes can be catalyzed by gold(I) catalysts. In a particular striking example propargylic alcohol **279** could be converted into bicyclic ketone **281** in good yields.

#### Heteroatom variants

The oxygen substituted versions of the DVCPR have been subject to more intense research in the covered time period than the corresponding nitrogen variants. In general there are two different modes for the heteroatom incorporation into the DVCPR, as part of the cyclopropane moiety or as part of one of the vinyl moieties. All variants are covered in the following.

**Scheme 35:**
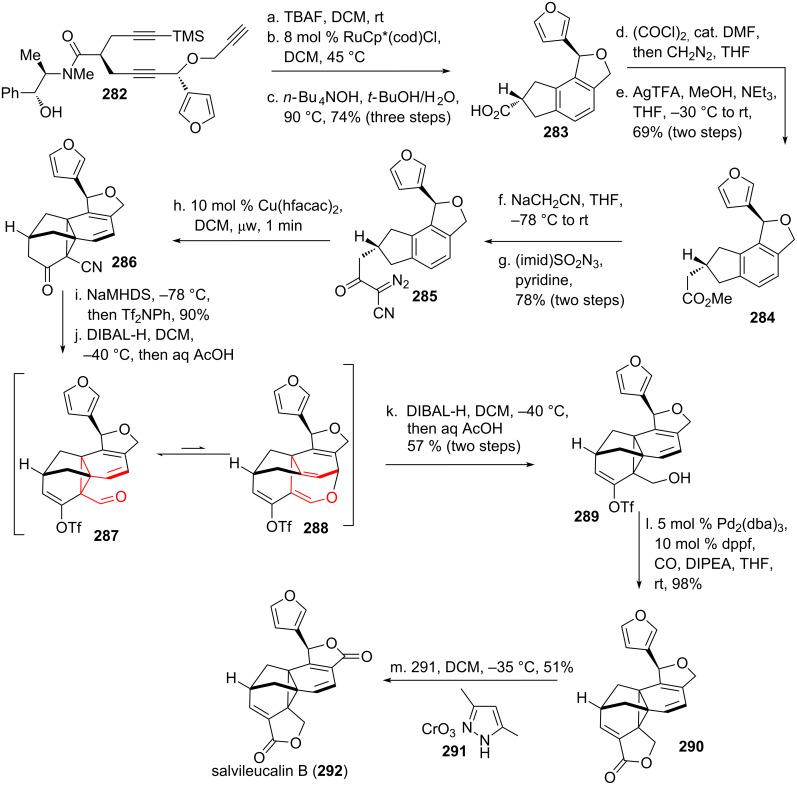
Reisman’s total synthesis of salvileucalin B, featuring an (undesired) vinylcyclopropyl carbaldehyde rearrangement.

The Reisman group [12,212] observed an intermediate vinylcyclopropane carbaldehyde rearrangement on their way towards the total synthesis of salvileucalin B (**292**, see Scheme 35) isolated from the plant *Salvia leucantha* [213]. Starting from enantiopure trialkyne **282**, desilylation was affected using TBAF, followed by ruthenium-catalyzed cycloisomerization [214] and cleavage of the chiral auxiliary to obtain tetracycle **283**. The carboxylic acid was converted into the corresponding acyl chloride, followed by addition of diazomethane and subsequent Arndt–Eistert homologation [215] to obtain methyl ester **284**. Claisen condensation furnished the intermediate β-cyano-ketone, which was subjected to diazotransfer conditions [216] to obtain **285**. This compound underwent smooth cyclopropanation with the adjacent benzene moiety to give *cis*-vinylcyclopropane cyanide **286**. Conversion of the ketone to the corresponding triflate followed by mono-reduction of the nitrile furnished *cis*-vinylcyclopropane carbaldehyde **287**, which underwent smooth, but undesired rearrangement to dihydrooxepin **288**. This rearrangement has been observed to be reversible [217]. Treatment with DIBAL-H reduced the carbaldehyde moiety selectively to give alcohol **289**. Lactonization was achieved upon Pd-catalyzed CO-insertion to give **290**. The desired natural product was obtained after oxidation of the tetrahydrofuran to the desired natural product salvileucalin B (**292**) using chromium trioxide·3,5-dimethylpyrazole [218].

#### Oxygen variants overview

**Scheme 36:**
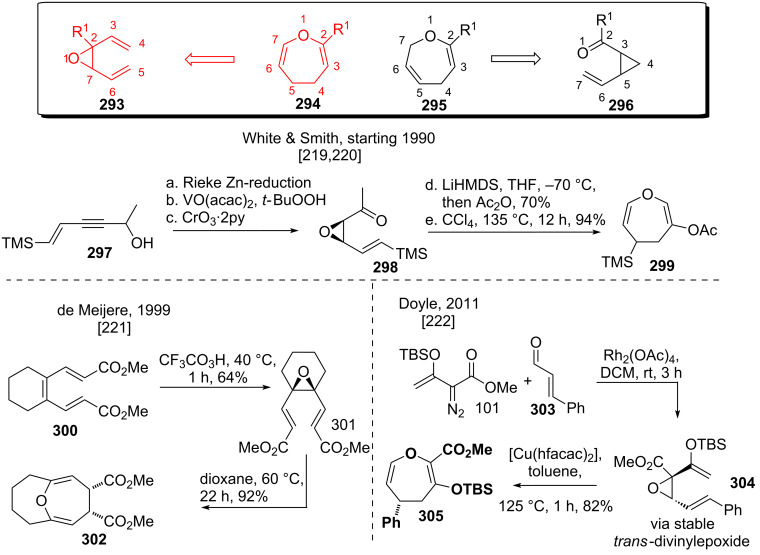
Studies on the divinylepoxide rearrangement.

The divinyloxirane rearrangement (**293** to **294**, see Scheme 36) has been investigated in detail by the groups of White and Smith [219–220]. The synthesis of the cyclization precursors started from enynes like **297**, beginning with *cis*-selective Rieke-Zn reduction. Epoxidation followed by oxidation furnished *cis*-vinylketone-epoxide **298**. Enolate formation and acetate trapping afforded an intermediate enol-acetate, which underwent high-yielding *cis*-divinylepoxide rearrangement at elevated temperatures to yield dihydrooxepine **299** in good yield. De Meijere and coworkers [221] investigated the selective epoxidation of hexatrienes like **300** to yield *cis*-divinylepoxide **301**. Gentle heating provided bridged bicycle **302**, containing two-*anti*-Bredt olefins in a stereospecific reaction. Doyle and coworkers [222] succeeded in preparing *trans*-divinylcyclopropanes like **304** via rhodium-catalyzed vinyldiazo decomposition (using compound **101**) and subsequent epoxide formation in the presence of cinnamon aldehyde (**303**). These rather stable epoxides (see **304**) were shown to undergo *trans*-*cis* isomerization followed by divinylepoxide rearrangement to give dihydrooxepine **305** at 125 °C. The necessary reaction temperature could be lowered using copper(II) catalysis, accelerating the reaction rate as well.

**Scheme 37:**
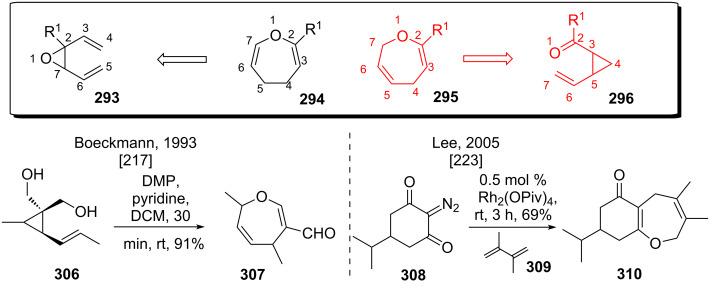
Studies on the vinylcyclopropanecarbonyl rearrangement.

The vinylepoxide–carbaldehyde rearrangement (**295** to **296**, see Scheme 37) has been investigated early on by the group of Boeckmann [217]. Dess–Martin periodinane oxidation of diol **306** resulted in a smooth rearrangement at ambient temperature, yielding formyldihydrooxepine **307**.

A modified approach has been investigated by the group of Lee, using transition metal mediated diazo decomposition [223]. The in situ generated rhodium-carbenoid formed upon diazo decomposition of **308** underwent cyclopropanation with butadienes like **309**. The resulting vinyl-epoxide ketone underwent rearrangement to give dihydrooxepine **310** in good yield. Vinylcyclopropane–cyclopentene rearrangement [13–14] has been shown to be the major competing side reaction in this case.

#### Nitrogen variants

**Scheme 38:**
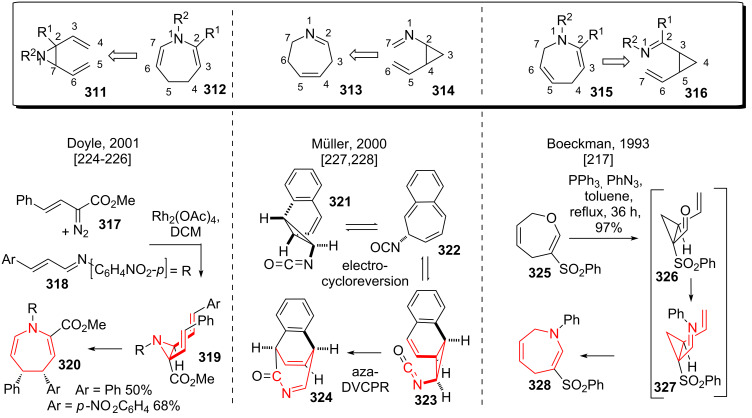
Nitrogen-substituted variants of the divinylcyclopropane rearrangement.

Three different approaches can be used to incorporate nitrogen into the DVCPR. The *cis*-divinylaziridine rearrangement (**311** to **312**, see Scheme 38) after rhodium-catalyzed aziridine formation between vinyl-diazo compound **317** and imine **318** has been found by Doyle [224–226]. Rearrangement occurred smoothly to give desired dihydroazepine **320**. Note that the authors suggest a transition state including an opened aziridine zwitterion. Alternatively, a cyclopropylamine can be used in an iminium ion type DVCPR rearrangement (**313** to **314**, see Scheme 38), or a cyclopropylcarbaldehyde can be condensed with an amine to an imine, which then undergoes the DVCPR rearrangement (**315** to **316**, see Scheme 38). Müller and coworkers [227–228] investigated the *trans*-vinylisocyanato–cyclopropyl rearrangement in great detail. Electrocycloreversion of initialy formed nitrene **321** leads to intermediate **322**, which can undergo electrocyclization to give the required *trans*-vinylisocyanatocyclopropane **323**, which underwent the desired rearrangement to give tricycle **324**. The group of Boeckman pioneered the heteroatom variants of the DVCPR, namely the vinylcyclopropane carbaldehyde–dihydrooxepine rearrangement [217], the vinylcyclopropane carbimine–dihydroazepine [217] rearrangement and the corresponding cyclobutane analogues [229–231]. A very stunning application [231] has been achieved using the Claisen reaction of dihydrooxepine **325** (see Scheme 38) to access vinylcyclopropane carbaldehyde **326**, which was in situ converted into the corresponding imine **327** using an aza-Wittig reaction. Subsequent vinylcyclopropane carbimine–dihydroazepine rearrangement furnished cyclic **328** in excellent yield.

## Conclusion

The divinylcyclopropane–cycloheptadiene rearrangement has been developed as a versatile method for the construction of seven-membered rings. The utilization in the total synthesis of both sesqui- and diterpenoid natural products as well as in the total synthesis of alkaloids and fatty acid-derived metabolites underlines its robustness and broad applicability. Contribution of the DVCPR as the key step to the solution of some of the most daunting synthetic challenges in present synthetic organic chemistry make it the tool of choice when constrained and highly substituted seven-membered rings are involved. The development of the formal [4 + 3]-cycloaddition (using a tandem cyclopropanation/DVCPR) has largely added to the scope of natural products that were synthesized using a DVCPR and shortened the overall synthetic routes.
